# Effectiveness of S-PRG Filler-Containing Toothpaste in Inhibiting Demineralization of Human Tooth Surface

**DOI:** 10.2174/1874210601812010811

**Published:** 2018-10-25

**Authors:** Bennett T. Amaechi, Hariyali Kasundra, Deepika Joshi, Azadeh Abdollahi, Parveez A. A. Azees, Linda O. Okoye

**Affiliations:** 1 Department of Comprehensive Dentistry, University of Texas Health Science Center, San Antonio, Texas, USA; 2 Department of Restorative Dentistry, Faculty of Dentistry’ College of Medicine, University of Nigeria Teaching Hospital, Ituku Ozalla, Enugu State, Nigeria

**Keywords:** Caries prevention, Fluoride, Ion-releasing, Surface pre-reacted glass-ionomer filler, Toothpaste, S-PRG filler, Dentifrice, Demineralization

## Abstract

**Objectives:**

Using an established pH-cycling caries model, the authors evaluated the effectiveness of toothpastes containing Surface Pre-reacted Glass-ionomer filler (S-PRG) in preventing tooth surface demineralization.

**Materials and Methods:**

210 tooth blocks were randomly assigned to seven experimental groups (30 blocks/group): no treatment (A), and toothpaste containing either NaF (B), 0 wt% S-PRG (C), 1 wt% S-PRG (D), 5 wt% S-PRG (E), 20 wt% S-PRG (F) or 30 wt% S-PRG (G). Groups were subjected to 14-day demineralization for development of early caries lesions using a pH-cycling caries model. Demineralization was assessed using Quantitative Light-induced Fluorescence (QLF) and Transverse Microradiography (TMR). All pairwise contrasts (between treatments) were tested using Analysis of Variance (ANOVA), and then Tukey’s HSD for multiple comparisons. All *p*-values are considered significant if <0.05.

**Results:**

With QLF, there was a significant (ANOVA; *p*<0.001) difference in mean percent fluorescence loss (∆F) observed among the groups. Relative to control, all S-PRG-containing toothpastes significantly (Tukey’s; *p*<0.0001) inhibited demineralization at varying percentages (48.6%, 61.3%, 67.4% and 69.8% reduction with S-PRG 1%, 5%, 20% and 30% respectively). Demineralization reduction was not significant with either NaF (15.6% reduction) or 0% S-PRG (-2.5% reduction *i.e.* 2.5% more demineralization than the Control) when compared to control group. Mineral loss assessed using TMR followed a similar trend as fluorescence loss.

**Conclusion:**

Toothpaste containing S-PRG filler can serve as an effective caries control tool. S-PRG filler-containing dentifrice to be more effective in preventing tooth demineralization than 1100 ppm fluoride provided as sodium fluoride.

## 1 INTRODUCTION

Despite being a preventable disease, dental caries remains a common chronic disease with global prevalence of 35% for all ages combined [1, 2]. Individuals at high caries risk still develop dental caries, despite numerous fluoride interventions available for the prevention and control of dental caries [3-6]. Thus search for other strategies that could work either synergistically with or better than fluoride in eradicating dental caries was recommended by the American Dental Association Council on Scientific Affairs Expert Panel on Non-fluoride Caries-Preventive Agents [7]. Following this recommendation, coupled with the paradigm shift in caries management towards preventive and minimal invasive dentistry, with initial caries lesions being managed non-operatively with therapeutic agents, numerous materials have been investigated, with a lot of them currently available in the market [5, 7, 8].

Among the new innovations for the management of dental caries is the three-layer bioactive Surface Pre-Reacted Glass ionomer filler (S-PRG), an active ingredient in commercially available GIOMER products (SHOFU Inc., Kyoto, Japan). S-PRG is engineered with the capability to release and recharge fluoride [9-11], in addition to the release of multiple other ions such as Strontium, Boron, Sodium, Aluminium and silicon dioxide ions at high concentrations [12]. This S-PRG ability is supported by the results of multiple studies on materials such as pits/fissure sealants, denture base resin, composite resins and resin barrier coats that contains S-PRG as an active ingredient [9-19]. These studies demonstrated that the multiple ion-releasing capacity endows on these materials the capability to inhibit demineralization of tooth tissue [14-17], promote remineralization of initial caries lesions [18] and reduce biofilm formation [13, 19]. While conventional fluoride (1100-1500 ppm) toothpastes cannot totally prevent tooth demineralization, combining strontium and fluoride enhances the efficacy of fluoride to promote remineralization compared to fluoride alone [20]. Thus, the aim of the present study was to investigate the effectiveness of toothpastes containing S-PRG to inhibit tooth surface demineralization, comparing it with a fluoride dentifrice containing 1100 ppm fluoride as sodium fluoride (NaF). Our first null hypothesis was that the treatments (NaF and S-PRG) would not significantly inhibit tooth surface demineralization (*i.e.* inhibit mineral loss and fluorescence radiance loss) relative to the control (no treatment). Our second null hypothesis was that the inhibition of tooth surface demineralization (prevention of mineral loss and fluorescence loss) by NaF and S-PRG would not be significantly different. Five concentrations of S-PRG were investigated to determine the most effective concentration.

## 2 MATERIALS AND METHODS

### 2.1 Preparation of Teeth and Experimental Grouping

Following the approval (IRB Approval #: HSC20080233N) of the Institutional Review Board of the University of Texas Health Science Center at San Antonio (UTHSCSA), unidentified sound human molar teeth freshly extracted either for orthodontic or third-molar impaction reasons, and appropriately disposed in various clinics of the UTHSCSA school of dentistry, were collected and autoclaved (120°C for 15 minutes). After sterilization, the teeth were cleansed of soft tissue debris, brushed with pumice slurry using an electric toothbrush (Braun Oral-B Vitality toothbrush, Proctor & Gamble, Cincinnati, Ohio, USA). 210 teeth without cracks, hypoplasia, white spot lesions and other malformations were selected following examination with a transilluminator. Water-cooled diamond wire saw (Walter Ebner, Switzerland) was used to cut off the roots of each tooth. Two coats of non-fluorescent, colorless, acid resistant nail varnish were painted on all surfaces of each tooth, except for an exposed enamel window (approximately 2 mm diameter) created on the buccal surface using discs of adhesive tape of uniform size. Each of the selected 210 teeth were randomly assigned to one of the seven experimental treatment groups, 30 teeth/group: (A) no treatment, and slurries of toothpastes containing either (B) 1100 ppm fluoride as 0.243% sodium fluoride (Crest™ Regular cavity protection, Proctor & Gamble, Cincinnati, Ohio, USA), (C) 0 wt% S-PRG filler, (D) 1 µm, 1 wt% S-PRG filler, (E) 1 µm, 5 wt% S-PRG filler, (F) 1 µm, 20wt% S-PRG filler or (G) 1 µm, 30wt% S-PRG filler. The toothpaste slurry was prepared by mixing 1 part dentifrice and 3 parts Distilled Deionized Water (DDW) using a laboratory stand mixer until homogenous. Using dental heavy duty putty, the 30 teeth in each group were embedded in oblong grooves carved inside a cylindrical acrylic rod that is attached to the cover of a 250-ml specially fabricated treatment tube. The seven groups were subjected to a 14-day demineralization for development of early caries, using the long established Featherstone laboratory pH cycling model accepted as a non-animal alternative to rat caries testing (Table **1**) [21, 22], while being treated with their respective product as described below.

### 2.2 Treatment Procedure

The pH cycling was performed as followed. Artificial saliva [23] containing MgCl_2_.6H_2_O (0.148 mmol/L), K_2_HPO_4_ (4.59 mmol/L), KH_2_PO_4_ (2.38 mmol/L), KCl (8.39 mmol/L), Calcium Lactate (1.76 mmol/L), Fluoride (0.05 ppm), Sodium Carboxymethylcellulose (2.25 mmol/L), and Methyl-4-hydroxybenzoate (13.14 mmol/L), with pH adjusted to 7.2 using KOH was used as the Remineralizing Solution (RS) in all treatment regimens, while an acidified buffer composed of 2.2 mmol/L KH_2_PO_4_, 2.2 mmol/L CaCl_2_), and 50 mmol/L Acetic Acid, with pH raised to 4.5 with KOH [24] was used as the Demineralizing Solution (DS) and serves as the acidic challenge medium. Fresh medium of either solutions were used on each treatment episode. Following 24 hours storage in RS, the cyclic treatment regimen for each day consisted of one 6-hour acid challenge in DS, two 2-minute toothpaste treatment periods, and then storage in RS for the rest of the time (Table **1**). For treatment, 200 ml of the treatment medium (RS, DS or toothpaste slurry) was placed into each 250-ml treatment tube. RS and DS treatments were magnetically stirred at 350 rpm, while the toothpaste slurry was static. All treatments were carried out in an incubator at 37ºC. The pH of each medium was measured once daily before treatment. The specimens were rinsed with running DDW after treatment with one medium, and dried with paper towel before immersion into the next agent. The daily regimen was repeated for 14 days before the termination of the experiment. Then the teeth were harvested and processed for demineralization assessment using Quantitative Light-induced Fluorescence (QLF) [25, 26] and Transverse Microradiography (TMR) [27].

### 2.3 QLF Imaging and Image Analysis

Following the pH-cycling treatment, demineralization of the tooth surface was assessed using QLF as follows. Using QLF clinical system, the fluorescent image of each tooth was captured and stored on the computer (PC) for later analysis. Prior to imaging, each tooth surface was dried by 5s air-drying with a dental air-water syringe in order to maintain a standardized hydration condition. The QLF system has an intra-oral camera device that is connected to a computer that bears the QLF software (Inspektor Research Systems BV, Amsterdam, The Netherlands). Image of the tooth is visualized and capture by illuminating the tooth with a blue-violet light with an intensity of 13 mW/cm^2^, which is generated by filtering white light from a special arc lamp (Philips bv, Eindhoven, The Netherlands) based on Xenon technology through a blue-transmitting bandpass filter (Philips bv, Eindhoven, The Netherlands) with peak intensity of λ = 370 nm and bandwidth of 80 nm. With a dental mirror providing a uniform illumination of the tooth, a color CCD-sensor (Sony LS-1P, Tokyo, Japan) that has a yellow-transmitting (λ ≥ 520 nm) filter (Philips bv, Eindhoven, The Netherlands) positioned in front of it (to filter out all reflected and back-scattered light) was used to record the fluorescent image of the tooth. A digitized image of the tooth surface was available for quantitative analysis with the QLF software [25, 26]. Once the fluorescent image of the tooth has been captured and recorded by the PC, analysis of the caries lesion (area of demineralization) can be initiated by a user-defined patch with borders placed on sound enamel surrounding the lesion. The sound fluorescence radiance values inside the patch are reconstructed through two-dimensional linear interpolation of sound enamel values on the patch borders [28]. The loss in fluorescence is determined by calculating the percentage difference between actual and reconstructed fluorescence surface. Any area with a fluorescence radiance drop of more than 5% is considered to be lesion [25, 26]. The QLF software automatically gives the value for the percentage fluorescence radiance loss, ΔF (%), with a simultaneous data storage [28, 29].

### 2.4 TMR Image Analysis

Demineralization was assessed with TMR after QLF imaging. To perform TMR analysis, a tooth slice (≈150 µm thick) was cut perpendicularly to the exposed enamel window in each tooth block. Using Adhesive Back 6 µm lapping film in a MultiPrep™ Precision Polishing machine (Allied High Tech, USA), each slice was polished on both sides to reduce the thickness of the slice to 100 µm (the appropriate thickness for TMR), and achieve a planoparallel surfaces. With a Phillips X-ray generator system (Panalytical, Amsterdam) set up for this purpose, the slices were microradiographed on type lA high resolution glass X-ray plates (Microchrome Technology, CA, USA). The plates were exposed for 10 minutes at an anode voltage of 20kV and a tube current of 10 mA, and then processed. Processing consisted of a 5 minute development in Kodak HR developer and 5 min fixation in Kodak Rapid-fixer before a final 30 minute wash period. The microradiographs were dried and examined with a Leica DMR optical microscope linked *via* a Sony model XC-75CE CCTV camera to a Computer housing the image analysis program (TMR2006 version 3.0.0.6, Inspektor Research, Amsterdam). Image of the microradiographs were analyzed under standard conditions of light intensity and magnification and processed, along with data from the image of the step wedge, by the TMR program. The computer program calculated the parameters of integrated mineral loss (∆z, vol%.µm) based on the work described by De Josselin de Jong *et al*. (1987) [27].

### 2.5 Statistical Analysis

Statistical analysis was performed using Stata 11.0 (StataCorp, College Station, TX) statistical software. The two null hypotheses were tested using Analysis of Variance (ANOVA). All pairwise contrasts (between treatments) were tested, and Tukey’s Honestly Significant Difference (HSD) test was used for multiplicity. The geometric mean ratios (*e.g.*, Treatment divided by Control) of both mineral loss and fluorescence loss are reported. With respect to control, ratios less than 1.0 indicate an improvement relative to control, and ratios greater than 1.0 indicate more mineral loss or fluorescence *i.e*., more severe lesions. All *p*-value was 2-sided and considered significant if less than 0.05.

## 3 RESULTS

In all treatment occasions, the pH of the toothpaste slurries ranged from 7.0-7.4. Analysis of variance (ANOVA) demonstrated a significant (*p*< 0.001) difference in mean values of both fluorescence loss (∆F) and mineral loss (∆z) observed among the groups (Table **2**).

With QLF measurements (∆F), all S-PRG filler-containing toothpastes significantly (Tukey’s; *p*<0.0001) inhibited demineralization at varying percentages relative to the control group (Table **2**). Demineralization reduction was not significant with toothpastes containing either NaF (15.6% reduction) or 0% S-PRG (-2.5% reduction *i.e.* 2.5% more demineralization than the control) when compared to control group (Table **2**). All comparisons of either NaF or 0% S-PRG toothpastes with all S-PRG filler-containing toothpastes were statistically significant (Tukey’s; *p*<0.0001) as well as the comparison of NaF with 0% S-PRG toothpastes. Thus, all toothpaste formulations with S-PRG filler (1%, 5%, 20% and 30%) achieved more than 20% (established for effectiveness) statistically significant difference with the standard fluoride toothpaste (NaF) in inhibiting demineralization (Table **2**). All comparisons of the S-PRG filler-containing toothpastes with each other are not statistically significant except the comparison of the toothpaste containing 1% S-PRG filler with those containing 20% and 30% that are significant (Tukey’s; *p*<0.0001). While S-PRG filler 1%, 5%, 20% and 30% had 0.51, 0.38, 0.33 and 0.31 times the fluorescence loss of the control, respectively, S-PRG filler 0% had almost equal (1.02 times) fluorescence loss as the control group and NaF had 0.84 times the fluorescence loss of the control. Similar result trend was observed with mineral loss (∆Z) measured using TMR (Table **2**). Figs. (**1** and **2**) respectively show the representative QLF images and TMR microradiographs from the experimental groups. In Fig. (**1**), the demineralization (whitespot lesions) in samples treated with S-PRG filler 5%, 20% and 30% was not visible by either QLF or visual examinations when the tooth surface is wet indicating non-clinical stage of caries development (International Caries Detection and Assessment System- ICDAS score 1). This is also reflected in their representative TMR microradiographs in Fig. (**2**), in which the subsurface demineralizations are very faint.

## 4 DISCUSSION

With the growing need for materials that can enhance the effect of fluoride or even work better than fluoride to eradicate persistent prevalence of both primary and secondary dental caries either as a preventive or therapeutic (remineralizing) agent, the present study investigated if surface pre-reacted glass ionomer fillers incorporated into toothpaste can effectively inhibit the demineralization of tooth tissue. This was compared with a non-prescription fluoride dentifrice containing 1100 ppm fluoride as NaF. Also investigated was the concentration of the S-PRG filler in toothpaste that would give optimal or adequate effectiveness for caries prevention. Effectiveness, evaluated by Transverse Microradiography (TMR) [27] and Quantitative Light-induced Fluorescence (QLF) [25, 26], was considered established if at least 20% statistically significant difference is observed between the S-PRG filler-containing toothpastes and the fluoride toothpaste for any one measurement method. These investigations were conducted using a lesion progression model (Caries prevention model) of the pH-cycling caries model (Table **1**). A pH-cycling model serves as a bridge to *in vivo* caries studies as they mirror clinical conditions, where demineralization and remineralization alternate constantly (*i.e*. pH cycling) and are only interrupted during the very short period of application of investigational products, such as toothpaste or mouthrinse. The pH-cycling model used in the present study is the long established Featherstone laboratory pH cycling model (Table **1**), which was developed and accepted as a non-animal alternative to animal caries reduction test (which is considered the “Gold Standard”) required by the Food & Drug Administration (FDA) for demonstration of efficacy of “Anticaries dentifrice product formulations” for over-the-counter human use [21, 22]. Following 14-day treatment, the samples were first examined visually for evidence of demineralization (whitespot lesion formation). It was observed that the whitespot (early caries) lesions on samples treated with toothpastes containing 5%, 20%, and 30% S-PRG filler were not visible when the samples were wet, but faintly visible when the samples were dried for 5s using a dental air-water syringe. Thus it was decided to use two devices to quantitatively assess the amount of demineralization; a highly sensitive clinical device (QLF) and a laboratory technique (TMR). Quantitative Light-induced Fluorescence (QLF) has long been demonstrated to be highly sensitive in detecting a very early stage of caries development that are not visible by clinical visual examination (non-clinical stage), and has since been use in studies involving caries detection and quantification, dental erosion measurement, and monitoring of toothwhitening [26, 30-33]. The tooth tissue has natural fluorescence radiance, which is reduced by demineralization (loss of mineral) [25, 26]. The fluorescence radiance loss (∆F) measured by QLF has been shown to be directly proportional to the amount of mineral lost through demineralization. [28, 30, 31, 33-35]. Transverse Microradiography, on the other hand, is considered the ‘Gold Standard’ for laboratory direct quantification of mineral loss in tooth tissue [27, 34, 35].

Different concentrations (weight%) of S-PRG filler (0, 1, 5, 20, 30 wt%) in toothpaste were investigated against an untreated control group and a group treated with a commercially available fluoride toothpaste containing 1100 ppm fluoride as NaF. The findings of the present study rejected our two null hypotheses that the treatments (NaF and S-PRG) would not significantly inhibit demineralization relative to the control, and that the inhibition of demineralization by NaF and S-PRG would not be significantly different. All toothpaste formulations with active ingredients (NaF or S-PRG filler) inhibited demineralization to varying percentages (Table **2**); however, the reduction was only significant and also greater with the S-PRG filler-containing toothpaste when compared with the control group. This was not surprising considering that previous studies had demonstrated S-PRG filler-containing materials to inhibit demineralization of tooth tissue [14-17], and promote remineralization of initial caries lesions [18]. This caries prevention and reversal ability of S-PRG filler-containing materials was attributed to the long demonstrated multiple ions (Sr^2+^,Na^+^,BO_3_^3-^,Al^3+^, SiO_3_^2-^ and F^2-^) releasing capability of S-PRG fillers [9-19]. Although the demineralization reduction by NaF (1100 ppm fluoride) in the present study was not significant relative to the control group, the ability of different fluoride formulations to prevent dental caries by inhibiting tooth tissue demineralization has been established in several studies [36-39]. In the present study, not only that all toothpaste formulations with S-PRG filler as the active ingredient achieved the set effectiveness criterion of at least 20% statistically significant higher demineralization inhibition than the NaF toothpaste as measured by both the TMR and QLF (Table **2**), the S-PRG filler-containing toothpaste formulations were 33-55% more effective in reducing tooth tissue demineralization than NaF-containing toothpaste. This again may be attributed to the single ion (F^2-^) releasing capacity of the NaF while the S-PRG filler releases six ions including fluoride ions, all of which play varying roles that support the inhibition of demineralization [9-19]. It is believed that the daily 6-hourly exposure of the tooth samples to acidic challenge opened micropores on the tooth tissue for uptake of these multiple ions, especially Sr^2+^, BO_3_^3-^ and F^2-^ that increase the resistant of the tooth to acid demineralization. Besides, the caries inhibition action of fluoride is dose-dependent with the standard concentration (1100-1500 ppm) unable to provide higher caries prevention in high acidic challenge situation as seen in cases of poor oral hygiene conditions as simulated in the present study [40, 41].

It is pertinent to mention that among the S-PRG filler containing toothpastes, while the demineralization inhibition of 1% S-PRG filler was significantly lower than those of 20% and 30% (Table **2**), the reduction of demineralization by the 5% S-PRG filler was comparable (not significantly difference) to those of 20% and 30% while at the same time not significantly different from that of 1% S-PRG. Thus 5% S-PRG filler seems to be an optimal or rather an ideal concentration of S-PRG filler to be incorporated into the toothpaste to provide caries prevention comparable to higher concentrations. However, considering that the present *in vitro* study was conducted with intense demineralization challenge (daily 6-hourly acidic exposure) for only 14 days, yet 48-50% demineralization inhibition was achieved with the 1% S-PRG filler, it is strongly believed that this concentration (1%wt S-PRG filler) when used for a long term as a daily oral hygiene product, will offer caries prevention in low and moderate caries risk individuals, while 5% S-PRG may be used for the high caries risk individuals. This can be confirmed through randomized controlled clinical trial, which is recommended to be the next step in the development of S-PRG filler for use in dentifrices for caries control.

## CONCLUSION

The present study demonstrated the effectiveness of toothpaste containing S-PRG filler in inhibiting tooth demineralization; thus highlighting its potential as an effective caries control tool. The study further demonstrated S-PRG filler-containing dentifrice to be more effective in preventing tooth surface demineralization than fluoride provided as sodium fluoride.

## Figures and Tables

**Fig. (1):**
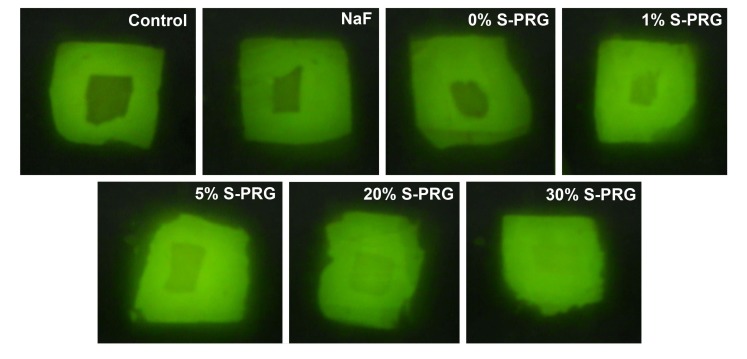
Representative QLF images from the experimental groups. (**A**) untreated, (**B**) 1100 ppm NaF, (**C**) 0 wt% S-PRG, (**D**) 1 wt% S-PRG, (**E**) 5 wt% S-PRG, (**F**) 20 wt% S-PRG, (**G**) 30 wt% S-PRG.

**Fig. (2):**
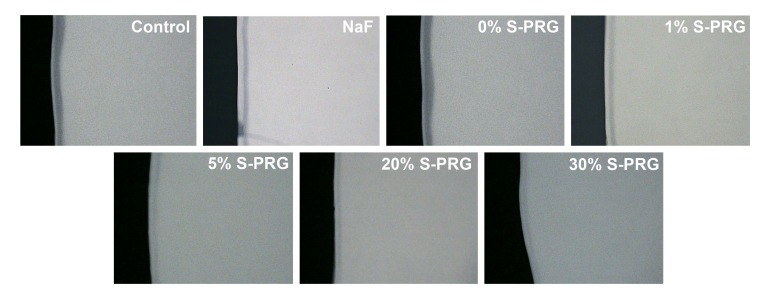
Representative microradiographs from the experimental groups. (**A**) untreated, (**B**) 1100 ppm NaF, (**C**) 0 wt% S-PRG, (**D**) 1 wt% S-PRG, (**E**) 5 wt% S-PRG, (**F**) 20 wt% S-PRG, (**G**) 30 wt% S-PRG.

**Table 1:** pH cycling treatment sequence for the experiment.

**Time**	**Treatment**
Day 1 is all-day storage in remineralization solution. Then, subsequent days’ treatments will be as follows	
2 min (starts 8:00 am) Approximately 1 hr to complete all groups.	Toothpaste treatment
Rinse with deionized distilled water	
6 hr (9:00 am – 3:00 pm)	Acid challenge (Demineralization)
Rinse with deionized distilled water	
2 min (starts 3:00 pm) Approximately 1 hr to complete all groups.	Toothpaste treatment
Rinse with deionized distilled water	
16 hrs (From 4:00 pm till 8:00 am next day)	Storage in Remineralization solution
Repeat for 13 additional Days (Human)	

**Table 2:** Mean (±SD) values of fluorescence loss (∆F) and mineral loss (∆Z) in each experimental groups and percentage inhibition of demineralization (as measured by ∆F and ∆Z) by individual intervention formulations relative to the untreated control group.

Treatment Groups	Mean ∆F ± SD	% Inhibition of Fluorescence Loss Relative to Control	Mean ∆Z ± SD	% Inhibition of Mineral Loss Relative to Control
Control (untreated)	23.33±4.59	**-**	933.2±183.6	**-**
NaF	19.69±2.82	15.6%	771.85±110.54	17%
0% S-PRG filler	23.91±3.53^b^	-2.5%	968.36±142.96^b^	-3.8%
1% S-PRG filler	12.00±2.47^a,b,c^	48.6%	466.88±96.08^a,b,c^	50%
5% S-PRG filler	9.03±1.39^a,b,c^	61.3%	360.79±55.53^a,b,c^	61.3%
20% S-PRG filler	7.60±1.09^a,b,c,d^	67.4%	304.04±43.6^a,b,c,d^	67.4%
30% S-PRG filler	7.05±0.37^a,b,c,d^	69.8%	274.95±14.43^a,b,c,d^	70.5%

^a^Significantly different from Control; ^b^Significantly different from NaF; ^c^Significantly different from 0% S-PRG filler; ^d^Significantly different from 1% S-PRG filler.

## LIST OF ABBREVIATIONS

ANOVA: = Analysis of Variance
DS: = Demineralizing Solution
DDW: = Distilled Deionized Water
FDA: = Food & Drug Administration
∆F: = Fluorescence Loss
HSD: = Honestly Significant Difference
ICDAS: = International Caries Detection and Assessment System
NaF: = Sodium Fluoride
QLF: = Quantitative Light-induced Fluorescence
RS: = Remineralizing Solution
S-PRG: = Surface Pre-reacted Glass-ionomer
TMR: = Transverse Microradiography
∆Z: = Mineral Loss
